# Evaluation of the Short-Term Effect of Tirzepatide on Liver Fibrosis Biomarkers Stratified by the Fibrosis-3 Index (FIB-3) in Patients With Type 2 Diabetes Mellitus

**DOI:** 10.7759/cureus.97279

**Published:** 2025-11-19

**Authors:** Daichi Yamamori, Takashi Kitao, Sachiko Masuda, Shimpei Koike, Tomoko Kaketaka, Yoshio Komoda, Erico Nakano, Eriko Konishi, Takeshi Ibata

**Affiliations:** 1 Diabetes and Endocrinology, Minoh City Hospital, Minoh, JPN; 2 Cardiology, Minoh City Hospital, Minoh, JPN

**Keywords:** apri, fib-3 values, liver fibrosis marker, masld, tirzepatide, type 2 diabetes mellitus

## Abstract

Aims

The primary objective of this study is to evaluate the short-term impact of tirzepatide on liver fibrosis markers, specifically the Fibrosis-3 index (FIB-3), Fibrosis-4 index (FIB-4), and aspartate aminotransferase-to-platelet ratio index (APRI), in patients with type 2 diabetes mellitus (T2DM). The secondary objective is to assess the correlations between FIB-3 and established fibrosis indices, including FIB-4 and APRI.

Methods

A total of 87 patients were stratified into two subgroups based on their FIB-3 score at the initiation of tirzepatide, following the FIB-3 classification: Group 1 (G1) (FIB-3 <1.5; n=68) and Group 2 (G2) (FIB-3 ≥1.5; n=19). We evaluated the changes in FIB-3, FIB-4, and APRI three months after the initiation of tirzepatide in each subgroup. Subsequently, we compared the changes (Δ) in FIB-3, FIB-4, and APRI scores across the two subgroups. Additionally, we investigated the baseline parameters and changes in these parameters correlated with Δ FIB-3. Correlation coefficients between FIB-3 and both FIB-4 and APRI were calculated.

Results

Only APRI exhibited a significant decrease in G1 (p=0.029), but FIB-3 (p<0.001), FIB-4 (p=0.005), and APRI (p<0.001) decreased significantly in G2. Δ FIB-3 (p=0.003), Δ FIB-4 (p=0.001), and Δ APRI (p<0.001) in G2 were significantly greater than those observed in G1. Δ FIB-3 was inversely correlated with baseline FIB-3 and positively correlated with Δ γ-glutamyl transpeptidase. FIB-3 demonstrated strong correlations with FIB-4 (r=0.81) and APRI (r=0.85).

Conclusions

In patients with T2DM, those with higher baseline FIB-3 values exhibited a more pronounced reduction in liver fibrosis markers during the short-term period following the initiation of tirzepatide therapy. FIB-3 exhibited robust associations with established fibrosis markers.

## Introduction

Fatty liver disease among individuals with type 2 diabetes mellitus (T2DM)

Previous studies have indicated that approximately half of non-alcoholic fatty liver disease (NAFLD) cases are comorbid with T2DM [1]. The nomenclature was revised to metabolic dysfunction-associated fatty liver disease (MASLD) in 2023 [2]. Accordingly, the terms MASLD and metabolic dysfunction-associated steatohepatitis (MASH) are used throughout this article.

MASLD is a form of fatty liver disease that occurs in the context of cardiometabolic risk factors (CMRFs), including obesity, T2DM, hypertension, and dyslipidemia. The prevalence of MASLD among individuals with T2DM exceeds 70%, with approximately half exhibiting a progressive form of the disease characterized by MASH and roughly one in five presenting with advanced liver fibrosis [3-5]. The presence of MASH markedly elevates the risk of liver-related complications, including cirrhosis, hepatocellular carcinoma (HCC), and overall mortality [6]. Timely detection and intervention in MASLD are essential to prevent progression to advanced liver fibrosis.

Usefulness of liver fibrosis markers in predicting prognosis

Histological evaluation via liver biopsy currently remains the gold standard for diagnosing MASH and staging liver fibrosis; however, it has notable limitations, including its invasive nature, risk of complications, sampling variability, and patient reluctance due to procedural invasiveness [7,8]. Therefore, the adoption of noninvasive tests (NITs) as essential tools for diagnosing, monitoring, and predicting the prognosis of MASLD has been promoted [9].

Common noninvasive methods for assessing liver fibrosis include the Fibrosis-4 index (FIB-4) [10] and the aspartate aminotransferase-to-platelet ratio index (APRI) [11]. FIB-4 has been recognized as a predictive marker for ischemic heart disease in individuals with fatty liver disease [12]. Elevated FIB-4, NAFLD fibrosis score (NFS), and APRI scores have been associated with an increased prevalence of heart failure [13]; moreover, liver fibrosis markers have been established as prognostic indicators for both hepatic and cardiovascular diseases.

Hepatic effects of tirzepatide

Tirzepatide is a novel antidiabetic agent that simultaneously targets the glucagon-like peptide-1 receptor (GLP-1R) and the glucose-dependent insulinotropic polypeptide receptor (GIPR), demonstrating superior glycemic control and weight-reducing effects compared to conventional GLP-1R agonists (GLP-1Ra) [14,15].

Tirzepatide administration has been reported to reduce FIB-4 scores [16]; however, as the FIB-4 formula incorporates age, age-specific cutoff values may be necessary, complicating its interpretation. To address this limitation, the Fibrosis-3 index (FIB-3) has been proposed as an age-independent marker of liver fibrosis [17]. To date, the impact of tirzepatide on FIB-3 has not been reported. This study aimed to evaluate the effect of tirzepatide administration on liver fibrosis markers, including FIB-3, in patients with T2DM.

## Materials and methods

Study design

This single-center, retrospective, observational study was conducted at Minoh City Hospital, Minoh, Japan, and used a prospectively maintained database. The study adhered to the ethical principles of the Declaration of Helsinki, and the protocol was approved by the Minoh City Hospital Ethics Committee (approval number: R0706B14). Informed consent was obtained using an opt-out procedure.

Subjects

**Figure 1:**
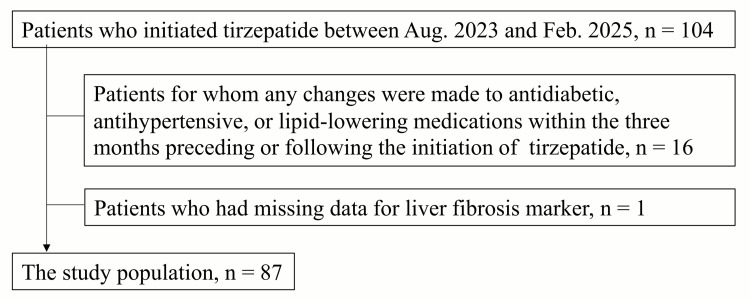
Flowchart of the study population

This study included 87 consecutive patients with T2DM who were initiated on tirzepatide between August 2023 and February 2025. We excluded patients younger than 20 years of age; patients with other chronic liver diseases, such as viral hepatitis and autoimmune hepatitis; patients for whom any changes were made to antidiabetic, antihypertensive, or lipid-lowering medications within the three months preceding or following the initiation of tirzepatide; patients who discontinued tirzepatide within three months after the initiation of tirzepatide; patients with incomplete data; and patients deemed unsuitable for the study by the attending physician. Figure 1 shows the flowchart of the study population.

Tirzepatide dosage

Tirzepatide was initiated at a dose of 2.5 mg once weekly and titrated to 5 mg weekly after four weeks, based on the physician's clinical judgment. In patients exhibiting an inadequate response after eight weeks, the dose was further escalated to 7.5 mg once weekly.

Data collection

We extracted demographic information, including age, sex, body weight, and clinical data such as medical history, comorbidities, medications, and laboratory data from medical records. Baseline laboratory data were evaluated at the initiation of the tirzepatide. The patients were monitored at least monthly by the attending physician, and blood tests were performed at each visit. FIB-3 [17], FIB-4 [10], and APRI [11] values at baseline and three months after the initiation of treatment were analyzed.

Classification of subjects

A total of 87 patients with T2DM were classified into two subgroups based on their FIB-3 at the initiation of tirzepatide in accordance with the FIB-3 classification, which is a tool for assessing the risk of liver fibrosis in patients with MASLD, as outlined in the previous studies [17]: Group 1 (G1) (low risk; FIB-3 <1.5; n=68) and Group 2 (G2) (moderate to high risk; FIB-3 ≥1.5; n=19). The FIB-3 was calculated using the following formula [17]: $\mathrm{FIB\text{-}3}=5\times\ln(\mathrm{AST\ (IU/L)})-2\times\ln(\mathrm{ALT\ (IU/L)})-0.18\times\mathrm{platelet}\ ( \times10^{4}/\mu\mathrm{L})-5$.

Outcomes

We evaluated the changes in FIB-3, FIB-4, and APRI three months after the initiation of tirzepatide in each subgroup. Subsequently, we compared the changes (Δ) in FIB-3, FIB-4, and APRI scores between the two subgroups. Then, we investigated the baseline parameters and changes (Δ) in the parameters correlated with Δ FIB-3. Additionally, we verified the correlation among liver fibrosis markers at baseline and the correlation among their changes three months after the initiation of tirzepatide. Finally, we evaluated the cutoff values of FIB-3 and FIB-4 for stratifying the risk of moderate to severe liver fibrosis.

Statistical analysis

Continuous variables are presented as medians (interquartile ranges), whereas categorical data are expressed as percentages. Statistical significance was assessed using the Wilcoxon signed-rank test and Mann-Whitney U test for continuous variables and Fisher's exact test for categorical variables. Univariate and multivariate analyses were performed using a linear regression model to assess baseline parameters and changes in parameters associated with Δ FIB-3. A multivariate analysis of baseline parameters associated with Δ FIB-3 was adjusted to account for age, body mass index (BMI), hemoglobin A1c (HbA1c), γ-glutamyl transpeptidase (γ-GTP), total bilirubin, and the FIB-3. Meanwhile, the multivariate analysis of changes in parameters associated with Δ FIB-3 was adjusted to account for Δ BMI, Δ estimated glomerular filtration rate (eGFR), Δ serum albumin, Δ HbA1c, Δ γ-GTP, and Δ total bilirubin. The correlation between liver fibrosis markers at baseline and the changes in these markers was assessed using the Pearson correlation coefficient. P-values of <0.05 were considered to indicate statistical significance. All analyses were conducted using the Bell Curve for Excel statistical software program (Version 4.08, Bell Curve for Excel, Social Survey Research Information Co., Ltd., Tokyo, Japan).

## Results

Patient characteristics at the initiation of tirzepatide between the two subgroups

Table 1 presents the patient characteristics between the two subgroups, classified based on their FIB-3 score at the initiation of tirzepatide, in accordance with the FIB-3 classification for assessing the risk of liver fibrosis. Fifty-six (64%) patients within the population transitioned from GLP-1Ra to tirzepatide, and 15 (17%) patients transitioned from dipeptidyl peptidase 4 inhibitor (DPP-4i) to tirzepatide. The median age was 60 (range: 49, 66) years, and 70% of the patients were male. The median body weight and BMI were 77 (range: 68, 88) kg and 27.5 (range: 24.8, 30.2) kg/m^2^. The median FIB-3, FIB-4, and APRI levels were -0.17 (range: -1.05, 1.10), 1.06 (range: 0.81, 1.51), and 0.26 (range: 0.20, 0.37), respectively. The median platelet count, aspartate aminotransferase (AST), alanine transaminase (ALT), and γ-GTP levels were 23.2 (range: 20.5, 27.5) ×10^4^/µL, 24 (range: 18, 32) IU/L, 25 (range: 18, 41) IU/L, and 29 (range: 21, 44) IU/L, respectively. At three months following tirzepatide initiation, 19 patients (22%) were receiving 2.5 mg, 63 patients (72%) were receiving 5 mg, and five patients (6%) were receiving 7.5 mg on a weekly basis.** **The median dose of tirzepatide three months after initiation was 5.0 (range: 5.0, 5.0) mg/week. No serious adverse events or treatment-emergent adverse events leading to discontinuation were observed after the initiation of tirzepatide. The administration rates of sodium-glucose cotransporter-2 (SGLT2) inhibitor, angiotensin-converting enzyme inhibitor (ACEi)/angiotensin II receptor blocker (ARB), fibrate, and thiazolidine were 86%, 67%, 28%, and 0%, respectively. Significant differences between the two subgroups were observed in the FIB-4, APRI, age, platelet count, AST levels, and γ-GTP levels.

**Table 1:** Baseline characteristics of the patients at the initiations of tirzepatide, stratified into two subgroups according to their FIB-3 Continuous variables are presented as median (interquartile range). Categorical data are presented as n (%). Tests for significance were conducted using the Mann-Whitney U test for continuous variables and Fisher's exact test for categorical data. ACEi: angiotensin-converting enzyme inhibitor; ALT: alanine transaminase; APRI: aspartate aminotransferase-to-platelet ratio index; ARB: angiotensin II receptor blocker; AST: aspartate aminotransferase; DPP-4i: dipeptidyl peptidase 4 inhibitor; eGFR: estimated glomerular filtration rate; FIB-3: fibrosis-3 index; FIB-4: fibrosis-4 index; GLP-1Ra: glucagon-like peptide-1 receptor antagonist; γ-GTP: γ-glutamyl transpeptidase; HbA1c: hemoglobin A1c; HDL: high-density lipoprotein; LDL: low-density lipoprotein; MRA: mineralocorticoid receptor antagonist; SGLT2i: sodium-glucose cotransporter 2 inhibitor

Clinical data	Overall (n=87)	FIB-3		z-value or v-value	p-value
		<1.5	≤1.5		
		G1 (n=68)	G2 (n=19)		
FIB-3	-0.17 (-1.05, 1.10)	-0.50 (-1.26, 0.26)	2.18 (1.54, 2.76)	-	-
FIB-4	1.06 (0.81, 1.51)	0.98 (0.77, 1.25)	2.12 (1.51, 2.36)	5.34	<0.001
APRI	0.26 (0.20, 0.37)	0.24 (0.18, 0.30)	0.54 (0.37, 0.65)	5.47	<0.001
Tirzepatide dosage (mg/week)	5.0 (5.0, 5.0)	5.0 (5.0, 5.0)	5.0 (2.5, 5.0)	1.49	0.14
Age (years)	60 (49, 66)	59 (48, 64)	68 (52, 74)	2.00	0.046
Male	61 (70%)	46 (68%)	15 (79%)	0.10	0.41
Body weight (kg)	77 (68, 88)	77 (68, 87)	80 (68, 88)	0.05	0.96
Body mass index (kg/m^2^)	27.5 (24.8, 30.2)	27.6 (24.8, 30.6)	27.1 (24.1, 29.8)	0.55	0.58
Systolic blood pressure (mmHg)	125 (115, 135)	125 (115, 136)	125 (118, 133)	0.31	0.76
Heart rate (bpm)	83 (75, 96)	83 (75, 96)	81 (72, 98)	0.38	0.70
Duration of diabetes (years)	11 (5, 17)	11 (5, 16)	15 (5, 26)	1.38	0.17
Hypertension	66 (76%)	48 (71%)	18 (95%)	0.16	0.12
Dyslipidemia	76 (87%)	61 (90%)	15 (79%)	0.13	0.25
Chronic kidney disease (eGFR <60)	23 (26%)	15 (22%)	8 (42%)	0.19	0.14
Laboratory data					
Hemoglobin (g/dL)	15.5 (14.2, 16.2)	15.4 (14.2, 16.1)	15.6 (13.5, 16.2)	0.07	0.94
Platelet (×10^4^/μL)	23.2 (20.5, 27.5)	23.9 (22.0, 29.5)	20.1 (17.0, 21.1)	4.36	<0.001
eGFR (mL/min/1.73 m^2^)	71 (59, 83)	74 (61, 87)	67 (45, 80)	1.64	0.10
Serum albumin (g/dL)	4.4 (4.2, 4.6)	4.3 (4.2, 4.5)	4.5 (4.3, 4.7)	0.84	0.40
Uric acid (mg/dL)	5.3 (4.7, 6.1)	5.3 (4.7, 6.0)	5.3 (4.7, 6.4)	0.51	0.61
HbA1c (%)	7.8 (6.9, 8.5)	8.0 (7.1, 8.8)	7.2 (6.8, 8.0)	1.62	0.11
AST (IU/L)	24 (18, 32)	21 (17, 27)	36 (26, 50)	4.15	<0.001
ALT (IU/L)	25 (18, 41)	25 (17, 38)	36 (20, 65)	1.63	0.10
γ-GTP (IU/L)	29 (21, 44)	26 (20, 42)	38 (25, 91)	2.80	0.005
Total bilirubin (mg/dL)	0.62 (0.43, 0.79)	0.60 (0.39, 0.74)	0.74 (0.55, 0.84)	1.46	0.14
LDL-cholesterol (mg/dL)	88 (73, 113)	88 (76, 119)	88 (67, 100)	1.43	0.15
Non-HDL-cholesterol (mg/dL)	117 (97, 144)	116 (98, 150)	117 (90, 127)	1.34	0.18
HDL-cholesterol (mg/dL)	50 (44, 59)	50 (44, 59)	49 (43, 61)	0.46	0.65
Triglyceride (mg/dL)	136 (104, 204)	135 (106, 207)	136 (80, 170)	0.71	0.48
C-reactive protein (mg/dL)	0.10 (0.06, 0.24)	0.09 (0.06, 0.25)	0.12 (0.09, 0.23)	0.68	0.50
Medication					
SGLT2i	75 (86%)	59 (87%)	16 (84%)	0.03	0.72
GLP-1Ra	56 (64%)	45 (66%)	11 (58%)	0.07	0.59
Biguanide	55 (63%)	45 (66%)	10 (53%)	0.12	0.29
Insulin	32 (37%)	26 (38%)	6 (32%)	0.06	0.79
DPP-4i	15 (17%)	9 (13%)	6 (32%)	0.20	0.09
Sulfonylurea/glinide	7 (8%)	7 (10%)	0 (0%)	0.16	0.34
Statin	67 (77%)	53 (78%)	14 (74%)	0.04	0.76
Ezetimibe	28 (32%)	21 (31%)	7 (37%)	0.05	0.78
Fibrate	24 (28%)	20 (29%)	4 (21%)	0.08	0.57
ACEi/ARB	58 (67%)	45 (66%)	13 (68%)	0.02	1.00
MRA	26 (30%)	21 (31%)	5 (26%)	0.04	0.78

Changes in variables three months after the initiation of tirzepatide in the two subgroups

**Figure 2:**
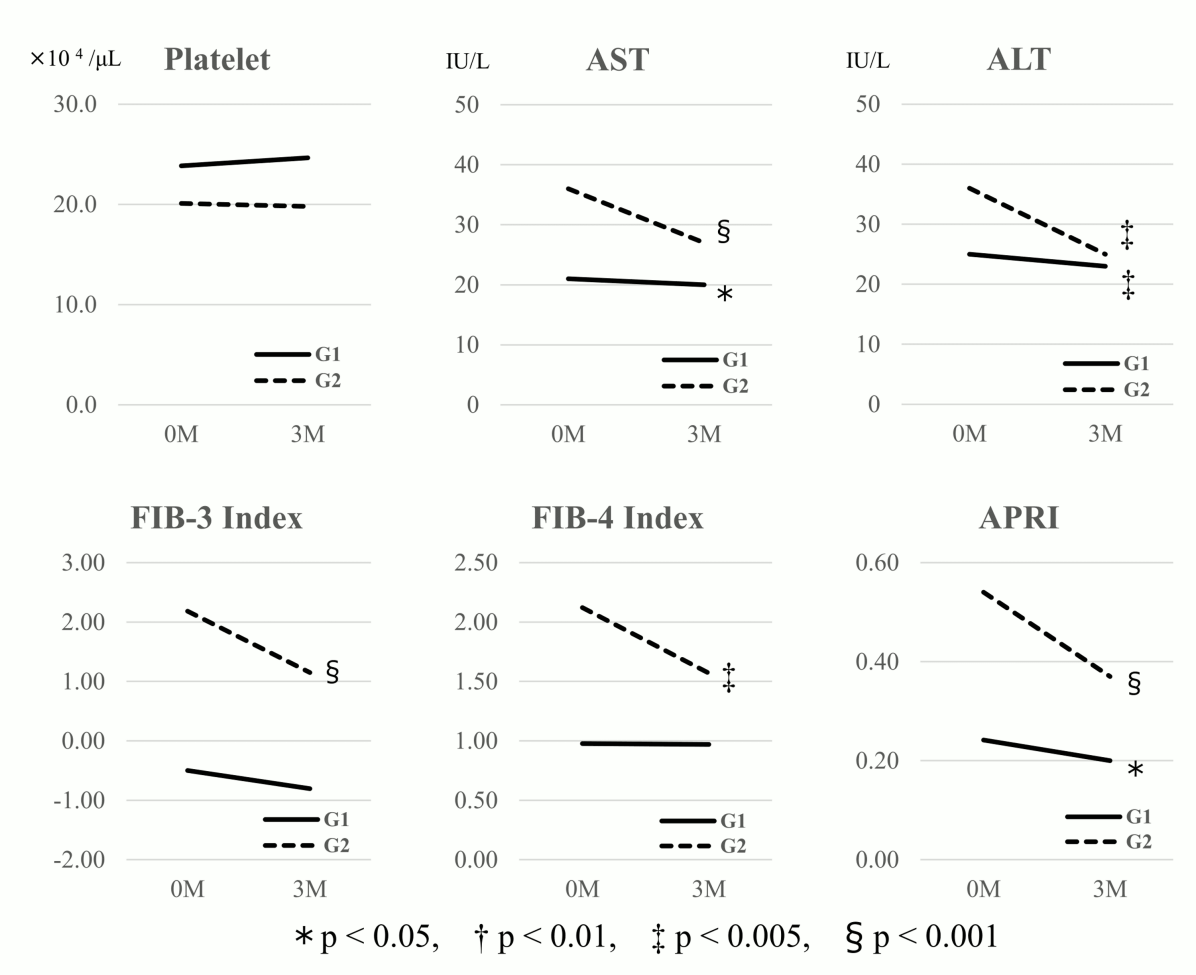
Variations in parameters three months after the initiation of tirzepatide between the two subgroups ALT: alanine transaminase; APRI: aspartate aminotransferase-to-platelet ratio index; AST: aspartate aminotransferase; FIB-3: fibrosis-3 index; FIB-4: fibrosis-4 index

Table 2 presents the changes in variables three months after the initiation of tirzepatide. Overall, significant reductions were observed in body weight, BMI, eGFR, uric acid, HbA1c, AST, ALT, γ-GTP, low-density lipoprotein cholesterol (LDL-C), non-high-density lipoprotein cholesterol (non-HDL-C), triglycerides, FIB-3, and APRI, whereas hemoglobin and serum albumin significantly increased. The AST, ALT, and APRI were significantly reduced in both G1 and G2, whereas FIB-3 and FIB-4 displayed significant reductions only in G2 (Figure 2).

**Table 2:** Variations in parameters three months after the initiation of tirzepatide Variables are presented as median (interquartile range). Tests for significance were conducted using the Wilcoxon signed-rank test. ALT: alanine transaminase; APRI: aspartate aminotransferase-to-platelet ratio index; AST: aspartate aminotransferase; eGFR: estimated glomerular filtration rate; FIB-3: fibrosis-3 index; FIB-4: fibrosis-4 index; γ-GTP: γ-glutamyl transpeptidase; HbA1c: hemoglobin A1c; HDL-C: high-density lipoprotein cholesterol; LDL-C: low-density lipoprotein cholesterol

Clinical data	Overall (n=87)				FIB-3							
					<1.5				≤1.5			
					G1 (n=68)				G2 (n=19)			
	0M	3M	z-value	p-value	0M	3M	z-value	p-value	0M	3M	z-value	p-value
Body weight (kg)	77 (68, 88)	75 (67, 85)	6.09	<0.001	77 (68, 87)	74 (67, 86)	5.17	<0.001	80 (68, 88)	80 (70, 84)	3.30	<0.001
Body mass index (kg/m^2^)	27.5 (24.8, 30.2)	26.9 (23.7, 29.8)	6.20	<0.001	27.6 (24.8, 30.6)	26.6 (23.7, 29.9)	5.19	<0.001	27.1 (24.1, 29.8)	27.2 (23.3, 29.5)	3.46	<0.001
Systolic blood pressure (mmHg)	125 (115, 135)	125 (110, 133)	1.36	0.17	125 (115, 136)	125 (110, 133)	0.72	0.47	125 (118, 133)	124 (112, 130)	1.73	0.08
Heart rate (bpm)	83 (75, 96)	86 (72, 94)	0.84	0.40	83 (75, 96)	86 (74, 96)	0.19	0.85	81 (72, 98)	77 (59, 93)	1.40	0.16
Laboratory data												
Hemoglobin (g/dL)	15.5 (14.2, 16.2)	15.5 (14.4, 16.3)	2.04	0.041	15.4 (14.2, 16.1)	15.4 (14.4, 16.3)	1.38	0.17	15.6 (13.5, 16.2)	16.0 (14.0, 16.7)	1.83	0.07
Platelet (×10^4^/μL)	23.2 (20.5, 27.5)	23.3 (19.8, 27.0)	1.11	0.27	23.9 (22.0, 29.5)	24.7 (21.9, 27.8)	0.45	0.65	20.1 (17.0, 21.1)	19.8 (18.0, 22.8)	1.69	0.09
eGFR (mL/min/1.73 m^2^)	71 (59, 83)	70 (54, 83)	3.28	0.001	74 (61, 87)	71 (60, 85)	3.16	0.002	67 (45, 80)	62 (43, 82)	1.15	0.25
Serum albumin (g/dL)	4.4 (4.2, 4.6)	4.4 (4.2, 4.7)	2.81	0.005	4.3 (4.2, 4.5)	4.5 (4.2, 4.7)	3.01	0.003	4.5 (4.3, 4.7)	4.5 (4.1, 4.7)	0.65	0.51
Uric acid (mg/dL)	5.3 (4.7, 6.1)	5.1 (4.3, 6.0)	2.09	0.037	5.3 (4.7, 6.0)	5.1 (4.2, 5.9)	2.02	0.043	5.3 (4.7, 6.4)	5.5 (4.7, 6.1)	0.68	0.49
HbA1c (%)	7.8 (6.9, 8.5)	6.7 (6.3, 7.4)	7.25	<0.001	8.0 (7.1, 8.8)	6.8 (6.3, 7.6)	6.56	<0.001	7.2 (6.8, 8.0)	6.5 (6.2, 7.3)	3.10	0.002
AST (IU/L)	24 (18, 32)	21 (17, 26)	3.74	<0.001	21 (17, 27)	20 (16, 24)	2.05	0.041	36 (26, 50)	27 (19, 34)	3.37	<0.001
ALT (IU/L)	25 (18, 41)	23 (16, 37)	4.07	<0.001	25 (17, 38)	23 (16, 34)	2.85	0.004	36 (20, 65)	25 (13, 51)	3.23	0.001
γ-GTP (IU/L)	29 (21, 44)	24 (16, 38)	5.13	<0.001	26 (20, 42)	22 (15, 34)	3.65	<0.001	38 (25, 91)	27 (20, 55)	3.62	<0.001
Total bilirubin (mg/dL)	0.61 (0.42, 0.79)	0.60 (0.43, 0.71)	0.27	0.79	0.60 (0.39, 0.74)	0.55 (0.40, 0.67)	0.96	0.34	0.74 (0.55, 0.84)	0.71 (0.56, 0.90)	1.41	0.16
LDL-cholesterol (mg/dL)	88 (73, 113)	75 (64, 103)	3.83	<0.001	88 (76, 119)	76 (64, 104)	3.60	<0.001	88 (67, 100)	72 (57, 96)	1.37	0.17
Non-HDL-cholesterol (mg/dL)	117 (97, 144)	104 (82, 129)	4.40	<0.001	116 (98, 150)	107 (85, 133)	3.95	<0.001	117 (90, 127)	92 (78, 121)	2.01	0.044
HDL-cholesterol (mg/dL)	50 (44, 59)	51 (43, 59)	0.66	0.51	50 (44, 59)	50 (43, 59)	0.47	0.63	49 (43, 61)	54 (46, 63)	0.36	0.72
Triglyceride (mg/dL)	136 (104, 204)	117 (90, 177)	2.86	0.004	135 (106, 207)	118 (93, 189)	2.35	0.019	136 (80, 170)	117 (80, 150)	1.77	0.08
C-reactive protein (mg/dL)	0.10 (0.06, 0.24)	0.08 (0.04, 0.17)	1.77	0.08	0.09 (0.06, 0.25)	0.08 (0.04, 0.17)	1.43	0.15	0.12 (0.09, 0.23)	0.07 (0.03, 0.18)	1.22	0.22
Liver fibrosis marker												
FIB-3	-0.17 (-1.05, 1.10)	-0.20 (-1.49, 0.93)	2.77	0.006	-0.50 (-1.26, 0.26)	-0.81 (-1.86, 0.26)	1.17	0.24	2.18 (1.54, 2.76)	1.15 (0.24, 1.82)	3.38	<0.001
FIB-4	1.06 (0.81, 1.51)	1.04 (0.76, 1.47)	1.60	0.11	0.98 (0.77, 1.25)	0.97 (0.73, 1.25)	0.02	0.99	2.12 (1.51, 2.36)	1.57 (1.22, 2.28)	2.82	0.0048
APRI	0.26 (0.20, 0.37)	0.21 (0.17, 0.32)	3.98	<0.001	0.24 (0.18, 0.30)	0.20 (0.17, 0.27)	2.18	0.029	0.54 (0.37, 0.65)	0.37 (0.30, 0.45)	3.62	<0.001

**Figure 3:**
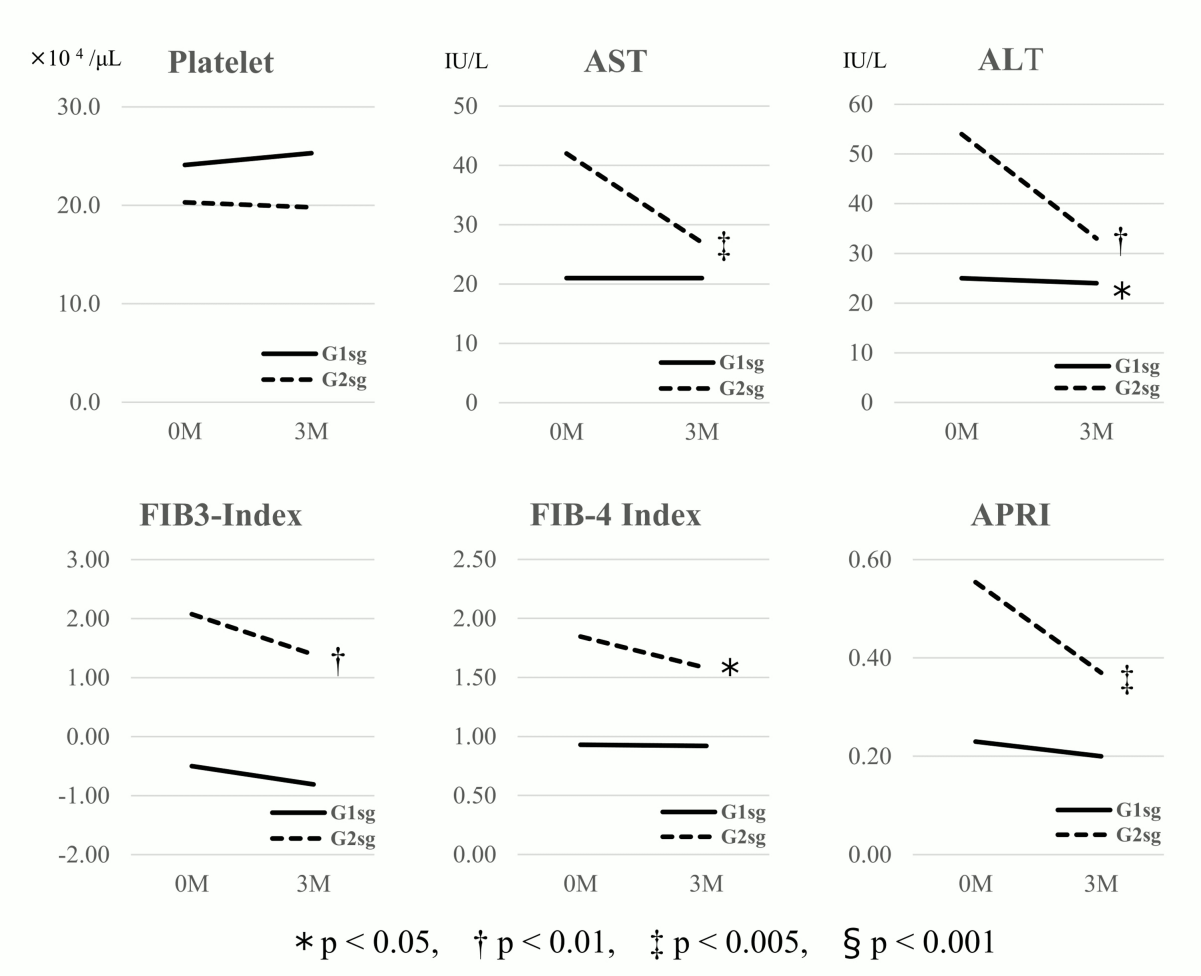
Variations in parameters between the two subgroups among patients with the switch from GLP-1Ra to tirzepatide ALT: alanine transaminase; APRI: aspartate aminotransferase-to-platelet ratio index; AST: aspartate aminotransferase; FIB-3: fibrosis-3 index; FIB-4: fibrosis-4 index; GLP-1Ra: glucagon-like peptide-1 receptor antagonist; G1sg: group 1 subgroup; G2sg: group 2 subgroup

A similar analysis was conducted in the G1 subgroup (G1sg; n=45) and the G2 subgroup (G2sg; n=11) among patients (n=56) who were switched from GLP-1Ra to tirzepatide, revealing significant reductions in liver enzymes and fibrosis markers in G2sg (Table 3 and Figure 3). All four groups (G1, G2, G1sg, and G2sg) showed significant reductions in body weight, although the absolute weight loss in each group was modest, ranging from 1 to 2 kg, with a corresponding weight loss rate of 1-3%.

**Table 3:** Variations in parameters among patients with the switch from GLP-1Ra to tirzepatide Variables are presented as median (interquartile range). Tests for significance were conducted using the Wilcoxon signed-rank test. ALT: alanine transaminase; APRI: aspartate aminotransferase-to-platelet ratio index; AST: aspartate aminotransferase; FIB-3: fibrosis-3 index; FIB-4: fibrosis-4 index; G1sg: group 1 subgroup; G2sg: group 2 subgroup; γ-GTP: γ-glutamyl transpeptidase; HbA1c: hemoglobin A1c

Clinical data	Overall (n=56)				FIB-3							
					<1.5				≤1.5			
					G1sg (n=45)				G2sg (n=11)			
	0M	3M	z-value	p-value	0M	3M	z-value	p-value	0M	3M	z-value	p-value
Body weight (kg)	80 (68, 90)	77 (67, 87)	4.69	<0.001	78 (68, 90)	76 (67, 87)	3.94	<0.001	83 (73, 90)	82 (71, 86)	2.43	0.015
Body mass index (kg/m^2^)	28.1 (25.7, 30.6)	27.4 (23.8, 29.8)	4.78	<0.001	28.0 (25.4, 30.8)	27.0 (23.8, 29.8)	3.92	<0.001	28.7 (27.0, 30.4)	28.4 (23.8, 30.0)	2.70	0.007
Laboratory data												
Platelet (×10^4^/μL)	23.8 (21.1, 28.6)	24.3 (21.0, 27.3)	0.02	0.27	24.1 (22.9, 30.2)	25.3 (22.6, 29.5)	0.08	0.93	20.3 (15.8, 21.1)	19.8 (16.8, 21.7)	0.53	0.59
HbA1c (%)	7.8 (6.9, 8.5)	6.9 (6.3, 7.7)	5.44	<0.001	7.8 (7.1, 8.5)	6.9 (6.3, 7.8)	5.08	<0.001	7.5 (6.7, 8.0)	6.6 (6.1, 7.4)	2.05	0.040
AST (IU/L)	25 (19, 34)	22 (17, 27)	3.02	0.003	21 (18, 27)	21 (17, 25)	1.32	0.19	42 (26, 50)	27 (19, 37)	2.94	0.003
ALT (IU/L)	26 (20, 43)	24 (16, 38)	3.41	<0.001	25 (20, 38)	24 (17, 36)	2.30	0.021	54 (20, 70)	33 (13, 52)	2.76	0.006
γ-GTP (IU/L)	29 (22, 44)	24 (17, 37)	4.08	<0.001	27 (18, 39)	23 (16, 32)	2.97	0.003	62 (30, 91)	40 (21, 58)	2.80	0.005
Liver fibrosis marker												
FIB-3	-0.30 (-1.39, 1.06)	-0.20 (-1.63, 0.94)	1.73	0.08	-0.50 (-1.71, 0.10)	-0.81 (-2.09, 0.13)	0.42	0.67	2.07 (1.54, 2.86)	1.39 (0.24, 2.17)	2.76	0.006
FIB-4	1.01 (0.81, 1.44)	1.02 (0.74, 1.36)	0.64	0.52	0.93 (0.73, 1.12)	0.92 (0.68, 1.15)	0.69	0.49	1.85 (1.71, 2.20)	1.58 (1.22, 2.29)	2.31	0.021
APRI	0.26 (0.18, 0.37)	0.21 (0.17, 0.32)	2.85	0.004	0.23 (0.17, 0.29)	0.20 (0.16, 0.27)	1.12	0.26	0.55 (0.37, 0.65)	0.37 (0.30, 0.47)	2.93	0.003

Comparison of changes (Δ) in FIB-3, FIB-4, and APRI between the two subgroups

**Figure 4:**
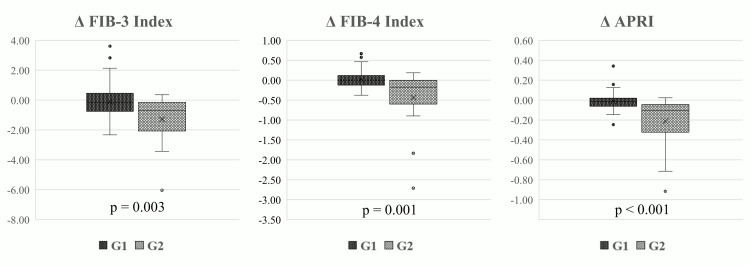
Comparison of changes in FIB-3, FIB-4, and APRI between the two subgroups APRI: aspartate aminotransferase-to-platelet ratio index; FIB-3: fibrosis-3 index; FIB-4: fibrosis-4 index

Table 4 and Figure 4 present a comparative analysis of changes (Δ) in FIB-3, FIB-4, and APRI between G1 and G2, revealing significant differences (p=0.003 for Δ FIB-3, p=0.001 for Δ FIB-4, and p<0.001 for Δ APRI).

**Table 4:** Comparison of changes in liver fibrosis markers between the two subgroups Variables are presented as median (interquartile range). Tests for significance were conducted using the Mann-Whitney U test. APRI: aspartate aminotransferase-to-platelet ratio index; FIB-3: fibrosis-3 index; FIB-4: fibrosis-4 index

Liver fibrosis marker	Overall (n=87)	FIB-3		z-value	p-value
		<1.5	≤1.5		
		G1 (n=68)	G2 (n=19)		
Δ FIB-3	-0.28 (-0.89, 0.35)	-0.17 (-0.76, 0.46)	-0.68 (-2.07, -0.15)	2.99	0.003
Δ FIB-4	-0.03 (-0.16, 0.10)	0.00 (-0.13, 0.12)	-0.17 (-0.60, 0.00)	3.29	0.001
Δ APRI	-0.03 (-0.08, 0.02)	-0.01 (-0.06, 0.02)	-0.10 (-0.32, -0.04)	4.11	<0.001

**Figure 5:**
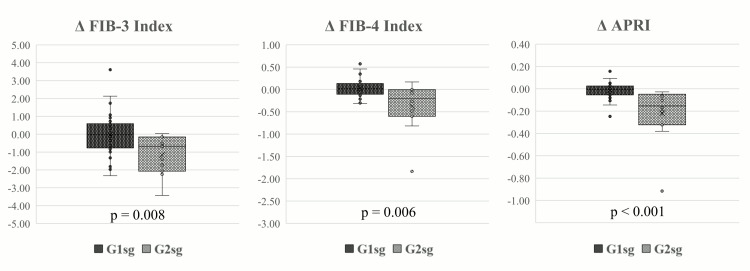
Comparison of changes in FIB-3, FIB-4, and APRI between the two subgroups among patients with the switch from GLP-1Ra to tirzepatide APRI: aspartate aminotransferase-to-platelet ratio index; FIB-3: fibrosis-3 index; FIB-4: fibrosis-4 index; G1sg: group 1 subgroup; G2sg: group 2 subgroup

Similarly, Table 5 and Figure 5 show a comparative analysis of changes in these markers between G1sg and G2sg, with significant differences observed (p=0.008 for Δ FIB-3, p=0.006 for Δ FIB-4, and p<0.001 for Δ APRI).

**Table 5:** Comparison of changes in liver fibrosis markers between the two subgroups among patients with the switch from GLP-1Ra to tirzepatide Variables are presented as median (interquartile range). Tests for significance were conducted using the Mann-Whitney U test. APRI: aspartate aminotransferase-to-platelet ratio index; FIB-3: fibrosis-3 index; FIB-4: fibrosis-4 index; G1sg: group 1 subgroup; G2sg: group 2 subgroup; GLP-1Ra: glucagon-like peptide-1 receptor antagonist

Liver fibrosis marker	Overall (n=56)	FIB-3		z-value	p-value
		<1.5	≤1.5		
		G1sg (n=45)	G2sg (n=11)		
Δ FIB-3	-0.18 (-0.93, 0.44)	-0.03 (-0.77, 0.59)	-0.68 (-2.07, -0.15)	2.65	0.008
Δ FIB-4	0.00 (-0.13, 0.12)	0.01 (-0.11, 0.13)	-0.20 (-0.60, 0.00)	2.77	0.006
Δ APRI	-0.03 (-0.07, 0.02)	0.00 (-0.05, 0.03)	-0.15 (-0.32, -0.05)	3.87	<0.001

Baseline parameters and changes (Δ) in parameters correlated with Δ FIB-3

Table 6 presents baseline parameters correlated with Δ FIB-3 three months after the initiation of tirzepatide. Δ FIB-3 was inversely correlated with baseline HbA1c (β=-0.184; p=0.031), γ-GTP (β=-0.293; p=0.007), total bilirubin (β=-0.169; p=0.041), and FIB-3 (β=-0.506; p<0.001).

**Table 6:** Baseline parameters correlated with Δ FIB-3 Tests for significance were conducted using linear regression model. β: standard partial regression coefficient; eGFR: estimated glomerular filtration rate; FIB-3: fibrosis-3 index; γ-GTP: γ-glutamyl transpeptidase; HbA1c: hemoglobin A1c

Variables	Univariate analysis		Multivariate analysis	
	β	p-value	β	p-value
Age (years)	0.194	0.07	0.293	0.001
Body mass index (kg/m^2^)	0.074	0.50	0.054	0.51
Duration of diabetes (years)	0.019	0.86	-	-
eGFR (mL/min/1.73 m^2^)	-0.078	0.47	-	-
HbA1c (%)	-0.040	0.71	-0.184	0.031
γ-GTP (IU/L)	-0.572	<0.001	-0.293	0.007
Total bilirubin (mg/dL)	-0.233	0.047	-0.169	0.041
Triglyceride (mg/dL)	0.039	0.72	-	-
FIB-3	-0.565	<0.001	-0.506	<0.001

Table 7 presents the changes (Δ) in parameters correlated with Δ FIB-3 three months after the initiation of tirzepatide. Δ FIB-3 demonstrated a significant positive correlation with Δ γ-GTP (β=0.491; p=0.001).

**Table 7:** Changes in parameters correlated with Δ FIB-3 Tests for significance were conducted using linear regression model. β: standard partial regression coefficient; eGFR: estimated glomerular filtration rate; γ-GTP: γ-glutamyl transpeptidase; HbA1c: hemoglobin A1c; LDL: low-density lipoprotein

Variables	Univariate analysis		Multivariate analysis	
	β	p-value	β	p-value
Δ body mass index (kg/m^2^)	0.229	0.042	0.021	0.88
Δ eGFR (mL/min/1.73 m^2^)	-0.218	0.042	0.097	0.43
Δ serum albumin (g/dL)	0.220	0.053	0.159	0.21
Δ HbA1c (%)	0.055	0.61	0.185	0.14
Δ γ-GTP (IU/L)	0.594	<0.001	0.491	0.001
Δ total bilirubin (mg/dL)	-0.043	0.72	-0.027	0.83
Δ LDL-cholesterol (mg/dL)	0.028	0.79	-	-
Δ triglyceride (mg/dL)	-0.028	0.80	-	-

Correlation among liver fibrosis markers at baseline and among their changes

**Figure 6:**
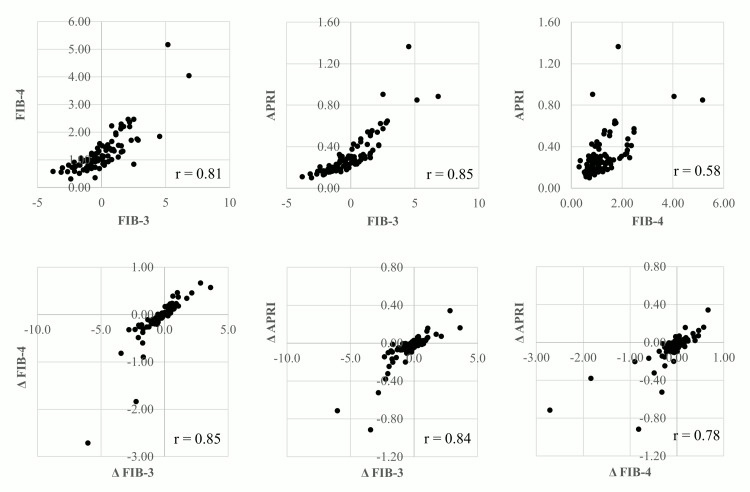
Correlation among liver fibrosis markers at baseline and among their changes APRI: aspartate aminotransferase-to-platelet ratio index; FIB-3: fibrosis-3 index; FIB-4: fibrosis-4 index

Figure 6 presents the correlations among liver fibrosis markers at baseline (upper row) and the correlations among their changes three months after the initiation of tirzepatide (lower row). Baseline FIB-3 showed strong correlations with both FIB-4 (r=0.81) and APRI (r=0.85), whereas the correlation between baseline FIB-4 and APRI was moderate (r=0.58). In contrast, Δ FIB-3 was strongly correlated with both Δ FIB-4 (r=0.85) and Δ APRI (r=0.84), and a strong correlation was also observed between Δ FIB-4 and Δ APRI (r=0.78).

Cutoff values of FIB-3 and FIB-4 for stratifying the risk of moderate to severe liver fibrosis

Table 8 presents the comparison of parameters between low and moderate to high risk of liver fibrosis according to the FIB-3 and FIB-4 classification. Within the liver fibrosis risk classification, baseline FIB-3, FIB-4, and APRI values were higher in the moderate- to high-risk group defined by FIB-3 (≥1.5) compared to those classified as moderate to high risk by FIB-4 (≥1.30).

**Table 8:** Comparison of parameters between low- and moderate- to high-risk groups of liver fibrosis according to the FIB-3 and FIB-4 classification Variables are presented as median (interquartile range). Tests for significance were conducted using the Wilcoxon signed-rank test. ALT: alanine transaminase; APRI: aspartate aminotransferase-to-platelet ratio index; AST: aspartate aminotransferase; FIB-3: fibrosis-3 index; FIB-4: fibrosis-4 index; γ-GTP: γ-glutamyl transpeptidase; HbA1c: hemoglobin A1c

Clinical data	Overall (n=87)	FIB-3		z-value	p-value	FIB-4		z-value	p-value
		<1.5	≤1.5			<1.30	≤1.30		
		G1 (n=68)	G2 (n=19)			S1 (n=55)	S2 (n=32)		
Laboratory data									
Platelet (×10^4^/μL)	23.2 (20.5, 27.5)	23.9 (22.0, 29.5)	20.1 (17.0, 21.1)	4.36	<0.001	24.9 (22.9, 30.1)	19.8 (17.2, 22.6)	5.75	<0.001
HbA1c (%)	7.8 (6.9, 8.5)	8.0 (7.1, 8.8)	7.2 (6.8, 8.0)	1.62	0.11	7.9 (7.1, 8.8)	7.5 (6.8, 8.5)	0.76	0.45
AST (IU/L)	24 (18, 32)	21 (17, 27)	36 (26, 50)	4.15	<0.001	21 (17, 29)	26 (20, 39)	2.15	0.031
ALT (IU/L)	25 (18, 41)	25 (17, 38)	36 (20, 65)	1.63	0.10	28 (20, 41)	22 (18, 42)	1.00	0.32
γ-GTP (IU/L)	29 (21, 44)	26 (20, 42)	38 (25, 91)	2.80	0.005	29 (23, 43)	30 (18, 66)	0.43	0.67
Total bilirubin (mg/dL)	0.61 (0.42, 0.79)	0.60 (0.39, 0.74)	0.74 (0.55, 0.84)	1.46	0.14	0.61 (0.39, 0.76)	0.62 (0.50, 0.83)	0.69	0.49
Liver fibrosis marker									
FIB-3	-0.17 (-1.05, 1.10)	-0.50 (-1.26, 0.26)	2.18 (1.54, 2.76)	-	-	-0.82 (-1.62, -0.17)	1.41 (0.68, 2.19)	6.74	<0.001
FIB-4	1.06 (0.81, 1.51)	0.98 (0.77, 1.25)	2.12 (1.51, 2.36)	5.34	<0.001	0.88 (0.73, 1.04)	1.71 (1.43, 2.20)	-	-
APRI	0.26 (0.20, 0.37)	0.24 (0.18, 0.30)	0.54 (0.37, 0.65)	5.47	<0.001	0.23 (0.17, 0.28)	0.33 (0.27, 0.55)	4.77	<0.001

## Discussion

Main findings

In patients with T2DM and a moderate to high risk of liver fibrosis as classified by FIB-3, liver enzyme levels, FIB-3, FIB-4, and APRI showed significant reductions from baseline three months after the initiation of tirzepatide. Moreover, the decreases in liver fibrosis markers were significantly greater than those observed in the low-risk group. The reduction in FIB-3 was more pronounced among individuals with higher baseline FIB-3 values and was positively correlated with decreases in γ-GTP levels. Both FIB-3 and Δ FIB-3 exhibited strong correlations with FIB-4, APRI, and their respective changes.

Temporal changes in liver enzymes after the initiation of tirzepatide

A significant reduction in AST and ALT levels was observed 26 weeks after initiating 15 mg of tirzepatide in patients with T2DM [18]. In patients with T2DM diagnosed with MASH and moderate to severe liver fibrosis confirmed by liver biopsy, AST and ALT levels declined in a time-dependent but dose-independent manner beginning at week 4 after the initiation of tirzepatide 5-10 mg, with further reductions observed beyond week 12 [19]. In the present study, regardless of baseline FIB-3 scores, significant reductions in body weight, HbA1c, AST, and ALT were observed three months following tirzepatide initiation (Table 2, Figure 2). These findings suggest that tirzepatide exerts its effects on hepatic enzymes within a relatively short timeframe.

Effects on liver fibrosis markers after the switch from GLP-1Ra to tirzepatide

In patients with T2DM and MASLD (n=54), significant reductions in body weight, HbA1c, ALT, AST, and FIB-4 (baseline mean: 1.30) were observed six months after the switch from GLP-1Ra to tirzepatide [16]. Conversely, in patients with T2DM (n=40) who switched from dulaglutide to tirzepatide, reductions in body weight, HbA1c, ALT, and AST were observed six months post-switch; however, no significant change in FIB-4 (baseline mean: 1.49) was reported [14]. Similarly, in this study, no reduction in FIB-4 (baseline median: 1.01) was observed among patients who switched from a GLP-1Ra to tirzepatide (n=56). However, among those with a baseline FIB-3 score ≥1.5 (G2sg; n=11), significant reductions in FIB-3, APRI, and FIB-4 (median baseline FIB-4: 1.85) were observed (Table 3, Figure 3). The baseline median FIB-4 values in patients with FIB-3 ≥1.5 in this study were higher than those reported in the two prior studies [14,16].

Association between hepatic effects of tirzepatide and baseline liver fibrosis severity

The reduction in ALT following the switch from dulaglutide to tirzepatide was more pronounced in patients with elevated baseline levels of AST, ALT, and γ-GTP, and FIB-4 also tended to decrease among those with higher baseline FIB-4 values [14]. Additionally, in patients with T2DM and a fatty liver index ≥60, the reduction in liver fat content (LFC) after tirzepatide administration was negatively correlated with baseline LFC levels [20]. In the present study, the reduction in FIB-3 was significantly greater in the group with high baseline FIB-3 values compared to the low baseline group (Table 4, Figure 4, Table 5, and Figure 5). Furthermore, multivariate analysis revealed a negative correlation between changes in FIB-3 and baseline FIB-3 values (Table 6). Consistent with previous reports, these findings suggest that the hepatoprotective effects of tirzepatide, including improvements in liver enzymes and fibrosis markers, are inversely associated with their respective baseline values.

Association between FIB-3 and FIB-4 and differences in cutoff values for intermediate liver fibrosis risk

In this study, liver fibrosis risk was stratified into low- and intermediate- to high-risk categories using FIB-3, which does not incorporate age into its calculation; however, significant differences in patient age were observed between the groups (Table 1). Since platelet count and ALT levels generally decline with age, FIB-3 may indirectly capture age-related hepatic changes, thereby complementing the influence of age on liver fibrosis [17]. Consequently, a strong correlation was observed between FIB-3 and FIB-4 (Figure 6). Moreover, within the liver fibrosis risk classification, baseline FIB-3, FIB-4, and APRI values were higher in the moderate- to high-risk group defined by FIB-3 (≥1.5) compared to those classified as moderate to high risk by FIB-4 (≥1.30) (Table 8). The moderate-risk classification according to FIB-3 is considered to indicate a higher risk level than a moderate-risk classification based on FIB-4. This finding suggests an association with a significant reduction in liver fibrosis markers and a more pronounced change in these markers among patients classified as moderate to high risk according to the FIB-3 score.

Possible effects of concomitant drugs on liver enzymes and liver fibrosis markers

Randomized controlled trials (RCTs) have demonstrated that pioglitazone, vitamin E [21], SGLT2 inhibitors [22], and GLP-1Ra [23] improve liver function in patients with hepatic conditions such as NAFLD (MASLD), NASH (MASH), and liver fibrosis. Although no participants in this study received pioglitazone, the high prevalence of SGLT2 inhibitor use (86%) raises the possibility of additive effects on liver enzyme levels. However, given the widespread use of SGLT2 inhibitors in patients with T2DM due to their established cardiorenal benefits, it may be challenging to isolate and evaluate the effects of tirzepatide in the absence of concomitant SGLT2 inhibitor use.

Indirect effects of incretins on hepatic function

GIPR is expressed in the pancreatic islet β-cells, adipocytes, hypothalamus, and brainstem [24]. However, GLP-1R is not expressed in the liver [25], suggesting that neither GLP-1R nor GIPR exerts direct effects on hepatocytes or hepatic stellate cells [26]. Consequently, the beneficial effects of GLP-1 and GIP on hepatic metabolism are regarded as largely indirect. In subcutaneous white adipose tissue, GIPR activation enhances blood flow, facilitates postprandial triglyceride uptake, and improves insulin sensitivity [27]. Enhanced lipid storage capacity in white adipose tissue may mitigate ectopic lipid accumulation in the liver. Animal studies have demonstrated that GIPR agonists can ameliorate insulin resistance independently of body weight changes [28] and enhanced insulin sensitivity in white adipose tissue is associated with reduced hepatic lipid deposition. Clinical trials have shown that tirzepatide enhances insulin sensitivity more effectively than conventional GLP-1Ra [29]. Administration of the dual GLP-1/GIP receptor agonist tirzepatide has been reported to improve hepatic fibrosis, the NAFLD activity score, and its individual components, including steatosis, lobular inflammation, and hepatocyte ballooning [30]. These findings suggest that tirzepatide's direct protective effects on peripheral tissues, particularly adipose tissue, may contribute to the amelioration of MASLD pathology. In this study, Δ BMI was not significantly associated with changes in the FIB-3 in multivariate analysis, whereas Δ γ-GTP was (Table 7), indicating that mechanisms beyond weight reduction may underlie the hepatic effects of tirzepatide.

Clinical implications

Our findings suggest that tirzepatide may be a therapeutic option for patients with T2DM and moderate to high liver fibrosis risk as indicated by the FIB-3 ≥1.5. In terms of clinical significance, we believe this study will help raise awareness of the importance of regularly assessing the risk of liver fibrosis in diabetes management. Furthermore, it suggests that cases with moderate to high risk of liver fibrosis that do not show improvement in liver fibrosis markers after tirzepatide administration should be considered high-risk cases for MASLD progression, prompting the consideration of intensified diabetes treatment, imaging examinations such as ultrasound, CT, MRI, and FibroScan, and further investigation through biopsy. We also believe it contributes to the standardization of the FIB-3 as a liver fibrosis marker that is not affected by age.

Limitations

Several limitations should be acknowledged in this study. First, the study employed a single-center, retrospective design with a limited observation period. Second, the sample size was small, particularly in the moderate- to high-risk group (G2). Third, the study was single-arm in nature and lacked a control group. Fourth, the evaluation was based solely on blood test results, without imaging or histopathological confirmation. To validate these findings and overcome the aforementioned limitations, future studies should incorporate larger sample sizes, adopt a multicenter, prospective, controlled trial design, and include a comprehensive assessment of liver fibrosis, including imaging modalities.

## Conclusions

In patients with T2DM, those with higher baseline FIB-3 values exhibited a more pronounced reduction in liver fibrosis markers during the short-term period following the initiation of tirzepatide therapy. FIB-3 exhibited robust associations with established fibrosis markers; however, the cutoff threshold for FIB-3 appeared higher than that for FIB-4.
